# Unmet environmental needs and unmet healthcare needs in a population of young adults with cerebral palsy: what the SPARCLE study tells us

**DOI:** 10.3389/fresc.2024.1294999

**Published:** 2024-02-02

**Authors:** Jonathan Rioual, Célia Perret, Catherine Arnaud, Nicolas Vidart d’Egurbide Bagazgoïtia

**Affiliations:** ^1^ UMR 1295 CERPOP, Inserm, Toulouse University III Paul Sabatier, Team SPHERE, Toulouse, France; ^2^ Clinical Epidemiology Unit, University Hospital, Toulouse, France

**Keywords:** cerebral palsy, unmet healthcare needs, environmental needs, impairments, young adults

## Abstract

**Introduction:**

Optimizing care for young adults with cerebral palsy is crucial for their physical and psychological well-being. The inadequacy of proximal environment may play a role in the provision of health services. The aim of this study is to explore the association between unmet environmental needs in the physical, social and attitudinal domains and unmet healthcare needs in four interventions: physiotherapy, occupational therapy, speech therapy and psychological counselling.

**Methods:**

Young adults with cerebral palsy were recruited in the SPARCLE3 European multicenter cross-sectional study. Healthcare needs and coverages were assessed using the Youth Health Care, Satisfaction, Utilization and Needs questionnaire. The need and availability of environmental factors in physical, social and attitudinal domains were collected using the European Adult Environment Questionnaire. Logistic regressions were conducted separately for each intervention to measure associations between unmet environmental needs and unmet healthcare needs.

**Results:**

We studied 310 young adults with cerebral palsy, with a mean age of 24.3 years; 37.4% could not walk independently, 51.5% had an IQ below 70, 34.2% had severe communication difficulties. The most commonly expressed need was physiotherapy (81.6% of participants). Unmet healthcare needs were reported by 20.9%, 32.4%, 40.3% and 49.0% of participants requiring physiotherapy, occupational therapy, psychological counselling and speech therapy, respectively. The physical environment was never significantly associated with unmet healthcare needs. In contrast, the social environment was significantly associated with unmet healthcare needs across all interventions, with odds ratios over 2.5, depending on the number of unmet needs and the nature of intervention needed. With regard to the attitudinal environment, when at least one unmet attitudinal environmental need was reported, the odds of also reporting an unmet healthcare need were of 3.68 for speech therapy and 3.77 for physiotherapy. The latter association was significant only for individuals with severe motor impairment.

**Discussion:**

Our results highlight the importance of the social and attitudinal environment in meeting healthcare needs in young adults with cerebral palsy. The lack of correlation between unmet healthcare needs and the physical environment suggests that it can be partly compensated for by social support.

## Introduction

The 2006 United Nations Convention on the Rights of Persons with Disabilities (1) underlines the obligation of Member States to take appropriate measures to ensure access for persons with disabilities to all aspects of life in order to promote their full participation in society on the basis of equal opportunities. Access to health services is one the major obstacles to equal opportunity for people with disabilities, particularly those with cerebral palsy.

Cerebral palsy (CP) is a complex condition that requires lifelong multidisciplinary care (2, 3). This care encompasses, among other things, access to specialized medical services, rehabilitation interventions, and psychological counselling. Optimizing and personalizing care is of paramount importance in meeting the specific needs of individuals with CP and maximizing their physical and psychological well-being (4). Medical advances have enabled individuals with CP to live longer, bringing their life expectancy closer to that of the general population (5). However, with improved survival come new health challenges. Young adults with CP face specific health issues, including a variety of clinical manifestations associated with CP (6) and early deterioration in health status (7). Compared with the general population, these young adults experience reduced walking ability, increased pain and fatigue, and mental health problems. It is therefore crucial to take account of these specific needs to provide them with appropriate care.

However, young adults with CP often have less access to health services in adulthood than they did in childhood (8) and face complex and varied barriers, such as a lack of specialized services tailored to adults or limited knowledge about adult CP among health professionals, that lead to gaps in continuity of care. As the International Classification of Functioning, Disability and Health (ICF) has defined disability since 2001 as resulting from a dynamic interaction between “body functions and structures” and “personal and environmental contextual factors”, we should also consider the inadequacy of the environment as a potential barrier to accessing health services.

There is little in-depth research specifically examining the overall impact of the environment on access to care for young adults with CP. The majority of existing studies have focused mainly on the medical and clinical aspects of care (9, 10), paying less attention to environmental factors likely to influence access to care. However, in the case of chronic conditions such as CP, it is often easier to modify an individual's environment than their abilities or bodily functions (11). It is therefore crucial to understand the extent to which inadequate physical, social, and attitudinal environments are associated with access to care among these young adults. To do this, we used unmet health needs as an indicator, which allows us to capture participants' actual experiences of accessing healthcare.

We aimed to explore the association between unmet environmental needs and unmet healthcare needs by focusing on four types of intervention: physiotherapy, occupational therapy, speech therapy, and psychological counselling. We hypothesized that the accumulation of unmet needs in the environment is associated with compounded unmet needs in health domains in young adults with CP.

## Methods

### Design and population

We used data from SPARCLE3, a European multicenter observational population-based cross-sectional study designed to investigate the impact of the environment on participation and quality of life of young adults with CP. The design and methods of the study have been described elsewhere (12). Briefly, the study population consisted of young adults diagnosed with CP as defined by the Surveillance of Cerebral Palsy in Europe (SCPE) network (13). They were born between 1991 and 1997, and were aged between 22 and 27 years at the time of data collection (2018–2020). They were randomly selected from regional registries in France, Sweden and Italy, and recruited from various sources in two other regions in Germany and Portugal.

### Data collection

Research assistants trained for the study visited the young adults with CP and conducted the interviews under identical conditions, with a logical flow and a fixed order for completing the questionnaires. Whenever possible, young adults completed the questionnaires themselves, with research assistants providing assistance as needed. When this was not possible, a relative or a personal assistant closely involved in their daily lives acted as a proxy.

Young adults with CP were asked about their healthcare needs, coverage and satisfaction of those needs, in various healthcare domains, using the short form of Youth Health Care, Satisfaction, Utilization and Needs (YHC-SUN) questionnaire (14). In this study, we considered only rehabilitation domains (physiotherapy, occupational therapy, speech therapy) and psychological counselling.

Information regarding the physical, social and attitudinal environment was collected using the European Adult Environment Questionnaire (EAEQ), a questionnaire developed as part of SPARCLE3, were which assesses the adequacy of 61 environmental factors (EF). Content density, diversity ratios and bandwidth index indicate that the EAEQ content links fairly well to the environmental classification of the ICF Core Set for adults with CP (15). Two types of information were available: the need of the EF and its availability in the event of need, or only its availability when the need was considered *a priori* to be common to all individuals. The responses were categorized as “unmet environmental need” when the response “Needed and not available” was provided, and “met environmental need” when the responses “Not needed” or “Needed and available” were ticked, depending on the item. In each domain of the environment, items related to access to care were selected *a priori* (9, 4 and 3 items in the physical, social, attitudinal domains, respectively, Supplementary Table S1).

Standardized information on impairments and comorbidities was collected: walking ability (using the Gross Motor Function Classification System, GMFCS (16) levels grouped into walkers (GMFCS I–III) and non-walkers (GMFCS IV–V)) and communication performance (Functional Communication Classification System, FCCS) (17), effective communication Yes (FCCS I–II)*/*No (FCCS III–V)). Intellectual ability was assessed with formal IQ testing or using an algorithm based on a set of questions to proxies (18), and thereafter categorized as <70/≥70. Young adults also reported their pain over the past week (not at all/once or twice/frequent) and seizures in the year predating interview [No (with or without medication)/Yes].

We also collected personal and family contextual factors: population size of area of residence (>200,000/3,000–200,000/<3,000 inhabitants), lifestyle [living alone independently/accompanied (family/partner)/ in care facilities], personal and parental highest education level completed (did not complete secondary education/secondary education/tertiary education), and perceived wealth (no or minimal financial difficulties/financial difficulties).

### Statistical analyses

Four subgroups of people were identified according to the health needs they reported to require for physiotherapy, occupational therapy, speech therapy, or psychological counselling (19). We first described the distribution of impairments, comorbidities, and contextual factors in the whole sample and in subgroups. The proportion of young adults who reported unmet environmental needs was estimated in the same subgroups. In addition, for each environmental domain, we plotted the proportion of individuals with unmet health needs against the number of unmet environmental needs (discrete variable) to determine categories, considering individuals with no unmet needs as the reference class, and a minimum of five individuals in each category.

Thereafter, separate analyses were carried out for physiotherapy, occupational therapy, speech therapy, and psychological counselling subgroups to assess the role of environmental factors on the satisfaction of healthcare needs. Bivariate comparisons relating the proportion of individuals with unmet health needs to socio-demographic and impairments characteristics were carried out. We then performed logistic regressions to estimate odds ratios (ORs) and their 95% confidence intervals (95% CI) which measured associations between unmet environmental needs in each domain and unmet physiotherapy/occupational therapy/speech therapy/psychological counselling need. All models were adjusted for country due to the recruitment (20) and policies for people with disabilities diversity across countries, and sex (21). Model 1 was consistently adjusted for GMFCS (22) and Model 2 for the size of the unit of residence and lifestyle (10, 23), as these were described in the literature as potential confounders. To identify potential additional confounding factors, two successive stepwise selections were then performed: the first added clinical variables (intellectual ability, seizures communication performance and frequency of pain) to Models 1, while the second included sociodemographic variables (education, wealth, and parental highest education level) in addition to Models 2, with the aim of minimizing the number of variables introduced into the models. Multivariate models were used to control for significant variables in separate analyses (*p* < 0.20) and these models were reduced using a descending step-by-step method with *p* < 0.05 as criterion for statistical significance. Participants with missing data for one or more variables in the different models were few, and they were excluded from the analyses.

Finally, sensitivity analyses were conducted. First, we considered only individuals who self-completed the questionnaires. Second, to examine the impact of each country on the results, with constraints of the small sample sizes, we excluded participants from each country one by one.

All analyses were performed using R (version 4.1.2, R Core Team, 2021).

## Results

Our sample consisted of 310 young adults with CP, with a mean age of 24.3 years [standard deviation (SD) 1.6 years], and a male-to-female ratio of 1.2. Table 1 shows impairments and socio-demographic characteristics. Briefly, 37.4% of individuals could not walk independently (GMFCS IV–V), 51.5% had an IQ < 70, and 34.2% had severe communication difficulties (FCCS III-V). Around 12% (11.7%) lived alone. In terms of healthcare needs, physiotherapy was the most frequently mentioned (81.6% of participants), followed by occupational therapy (47.7%), psychological counselling (40.0%), and speech therapy (31.6%). Of the participants who reported all rehabilitation needs (physiotherapy, speech therapy and occupational therapy, *n* = 72), 56.9% had a GMFCS IV–V, 66.7% had a FCCS level III to V, and a small proportion (4.2%) lived alone. Three quarters (75%) of those who reported a need for speech therapy, whether or not they expressed other needs, had not completed secondary education, compared with 58.0% in the total sample. The group of young adults who said they needed psychological counselling, alone or in combination with other needs, had less severe motor (33.1% with GMFCS IV–V) and cognitive (86.7% with an IQ < 70) impairments than the group as a whole. They were also more likely to live alone than participants as a whole (16.1%).

**Table 1:** Characteristics of young adults with CP across the entire sample and by health needs subgroups.

	All participants (*n* = 310)		Physiotherapy need (*n* = 253)		Occupational therapy need (*n* = 148)		Speech therapy need (*n* = 98)		Psychological counselling need (*n* = 124)	
Sex										
Male	171	55.2	133	52.6	79	53.4	54	55.1	69	55.6
Female	139	44.8	120	47.4	69	46.6	44	44.9	55	44.4
Age (years)										
22	52	16.8	46	18.2	23	15.5	21	21.4	20	16.1
23	54	17.4	42	16.6	32	21.6	18	18.4	26	21.0
24	66	21.3	57	22.5	37	25.0	23	23.5	26	21.0
25	64	20.6	47	18.6	27	18.2	21	21.4	23	18.5
26	42	13.5	35	13.8	13	8.8	10	10.2	16	12.9
27	32	10.3	26	10.3	16	10.8	5	05.1	13	10.5
Means (SD)	24.3	(1.57)	24.2	(1.58)	24.2	(1.53)	24.0	(1.46)	24.2	(1.57)
Impairment characteristics	N	%	n	%	n	%	n	%	n	%
GMFCS										
I–II–III	194	62.6	142	56.1	71	48.0	45	45.9	83	66.9
IV–V	116	37.4	111	43.9	77	52.0	53	54.1	41	33.1
Intellectual impairment										
≥70	127	48.5	94	44.3	43	33.3	12	15.0	54	48.6
<70	135	51.5	118	55.7	86	66.7	68	85.0	57	51.4
Seizures in the previous year										
No (with or without medication)	259	83.8	208	82.5	114	77.0	73	74.5	107	87.0
Seizures	50	16.2	44	17.5	34	23.0	25	25.5	16	13.0
FCCS		** **	** **	** **		** **		** **		** **
I–II	204	65.8	159	62.8	71	48.0	38	38.8	83	66.9
III–IV–V	106	34.2	94	37.2	77	52.0	60	61.2	41	33.1
Frequency of pain in previous week										
None	108	35.0	78	31.1	49	33.3	36	36.7	35	28.2
Once or twice	129	41.7	57	22.7	61	41.5	37	37.8	51	41.1
Frequent	72	23.3	116	46.2	37	25.2	25	25.5	38	30.6
Sociodemographic characteristics	N	%	n	%	n	%	n	%	n	%
Country										
France	82	26.5	69	27.3	36	24.3	29	29.6	39	31.5
Sweden	29	09.4	23	09.1	23	15.5	12	12.2	11	08.9
Italy	23	07.4	20	07.9	15	10.1	9	09.2	14	11.3
Germany	78	25.2	70	27.7	29	19.6	26	26.5	22	17.7
Portugal	98	31.6	71	28.1	45	30.4	22	22.4	38	30.6
Size of unit of residence (inhabitants)										
>200,000	107	34.7	84	33.3	43	29.3	27	27.6	38	30.9
3,000–200,000	145	47.1	120	47.6	76	51.7	48	49.0	62	50.4
< 3,000	56	18.2	48	19.0	28	19.0	23	23.5	23	18.7
Lifestyle										
Living alone	36	11.7	31	12.3	12	08.2	4	04.1	20	16.1
Living accompanied	242	78.3	195	77.4	110	74.8	80	82.5	93	75.0
In care facilities	31	10.0	26	10.3	25	17.0	13	13.4	11	08.9
Education level										
Did not complete secondary education	177	58.0	150	60.5	94	65.3	72	75.0	73	60.3
Secondary education	83	27.2	62	25.0	40	27.8	20	20.8	32	26.4
Tertiary education	45	14.8	36	14.5	10	06.9	4	04.2	16	13.2
Parental education level										
Did not complete secondary education	110	36.1	90	36.1	52	35.9	36	37.1	43	35.2
Secondary education	101	33.1	84	33.7	46	31.7	31	32.0	39	32.0
Tertiary education	94	30.8	75	30.1	47	32.4	30	30.9	40	32.8
Wealth										
No or minimal financial difficulties	234	77.2	185	74.0	98	68.1	67	70.5	91	76.5
Financial difficulties	69	22.8	65	26.0	46	31.9	28	29.5	28	23.5

CP, cerebral palsy; SD, standard deviation; GMFCS, Gross Motor Function Classification System; FCCS, Fonctionnal Communication Classification System.

 Table 2 shows the distribution of unmet environmental needs in the physical, social and attitudinal domains, for the whole sample and for each group that reported a healthcare need, while the responses for each EF are provided in Supplementary Table S2. The proportion of subjects reporting the highest number of unmet environmental needs per domain (4–9, 2–4 and 1–3 for the physical, social, attitudinal environments, respectively) is lowest for the social environment, overall and for each group of expressed healthcare needs. In the subgroup of individuals who reported requiring all three rehabilitation interventions, unmet needs were high: 73.2%, 38.9% and 40.8% of individuals with at least one unmet need in the physical, social, and attitudinal environments, respectively, compared to 62.1%, 27.3%, 32.7% for the same environmental domains in the whole sample. Individuals who reported a need for psychological counseling also had a higher prevalence of unmet environmental needs in all three domains compared to the whole population, with 66.9%, 32.3%, and 38.8% of individuals with at least one unmet need in each domain of environment.

**Table 2:** Distribution of unmet needs in the three environmental domains within the total population and by healthcare needs subgroup.

	All participants (*n* = 310)		Physiotherapy need (*n* = 253)		Occupational therapy need (*n* = 148)		Speech therapy need (*n* = 98)		Psychological counselling need (*n* = 124)	
	*N*	%	*n*	%	*n*	%	*n*	%	*n*	%
Physical environment (9 items)	** **	** **		** **		** **		** **	** **	** **
0 unmet need	116	37.9	76	30.4	41	27.9	25	26.0	41	33.1
1–3 unmet needs	114	37.3	101	40.4	59	40.1	39	40.6	51	41.1
4–9 unmet needs	76	24.8	73	29.2	47	32.0	32	33.3	32	25.8
Social environment (4 items)	** **	** **		** **		** **	** **	** **	** **	** **
0 unmet need	224	72.7	173	68.9	99	67.8	62	63.3	84	67.7
1 unmet need	68	22.1	62	24.7	38	26.0	29	29.6	30	24.2
2–4 unmet needs	16	05.2	16	06.4	9	06.2	7	07.1	10	08.1
Attitudinal environment (3 items)	** **	** **		** **		** **		** **	** **	** **
0 unmet need	204	67.3	165	66.5	90	61.6	59	61.5	74	61.2
1–3 unmet needs	99	32.7	83	33.5	56	38.4	37	38.5	47	38.8

The proportion of individuals who reported unmet healthcare needs was the lowest for physiotherapy (20.9%), followed by occupational therapy (32.4%), psychological counselling (40.3%), and speech therapy (49.0%). Table 3 shows to what extent the proportion of individuals with unmet healthcare needs varied according to nature and severity of impairments, country of residence and socio-demographic characteristics. We observed a lower proportion of people with unmet healthcare needs for all types of rehabilitation interventions in young adults with GMFCS IV–V compared to those with GMFCS I–III. Significant differences between countries were observed for physiotherapy unmet needs (from 7.1% of the participants in Germany to 31.0% in Portugal; *p* = 0.004) and occupational therapy unmet needs (from 4.1% in France to 44.4% in Portugal, *p* = 0.010).

**Table 3:** Distribution of healthcare needs met and unmet within the four subgroups of healthcare needs.

	Physiotherapy need (*n* = 253)					Occupational therapy need (*n* = 148)					Speech therapy need (*n* = 98)					Psychological counselling need (*n* = 124)				
	Met (*n* = 200)		Unmet (*n* = 53)			Met (*n* = 100)		Unmet (*n* = 48)			Met (*n* = 50)		Unmet (*n* = 48)			Met (*n* = 74)		Unmet (*n* = 50)		
	*n*	%	*n*	%	*p*	*n*	%	*n*	%	*p*	*n*	%	*n*	%	*p*	*n*	%	*n*	%	*p*
Sex					0.966					0.356					0.823					0.003
Male	105	78.9	28	21.1		56	70.9	23	29.1		27	50.0	27	50.0		33	47.8	36	52.2	
Female	95	79.2	25	20.8		44	63.8	25	36.2		23	52.3	21	47.7		41	74.5	14	25.5	
Age (years)					0.233					0.666					0.542					0.932
Means (SD)	24.2	(1.60)	24.5	(1.51)		24.1	(1.49)	24.2	(1.61)		23.9	(1.49)	24.0	(1.44)		24.2	(1.52)	24.2	(1.66)	
GMFCS					0.052					0.002					0.016					0.169
I–II–III	106	74.6	36	25.4		39	54.9	32	45.1		17	37.8	28	62.2		46	55.4	37	44.6	
IV–V	94	84.7	17	15.3		61	79.2	16	20.8		33	62.3	20	37.7		28	68.3	13	31.7	
Intellectual impairment					0.787					0.111					0.851					0.058
≥70	71	75.5	23	24.5		25	58.1	18	41.9		6	50.0	6	50.0		37	68.5	17	31.5	
<70	91	77.1	27	22.9		62	72.1	24	27.9		32	47.1	36	52.9		29	50.9	28	49.1	
Seizures in the previous year					0.108					0.991					0.082					0.150
No (with or without medication)	169	81.2	39	18.8		77	67.5	37	32.5		41	56.2	32	43.8		67	62.6	40	37.4	
Seizures	31	70.5	13	29.5		23	67.6	11	32.4		9	36.0	16	64.0		7	43.8	9	56.2	
FCCS					0.461					0.296					0.504					0.082
I–II	128	80.5	31	19.5		45	63.4	26	36.6		21	55.3	17	44.7		54	65.1	29	34.9	
III–IV–V	72	76.6	22	23.4		55	71.4	22	28.6		29	48.3	31	51.7		20	48.8	21	51.2	
Frequency of pain in previous week					0.553					0.479					0.416					0.036
None	60	75.0	20	25.0		30	61.2	19	38.8		17	47.2	19	52.8		16	45.7	19	54.3	
Once or twice	87	81.3	20	18.7		44	72.1	17	27.9		22	59.5	15	40.5		37	72.5	14	27.5	
Frequent	53	80.3	13	19.7		25	67.6	12	32.4		11	44.0	14	56.0		21	55.3	17	44.7	
																				
																				
Country					0.004					0.010					0.063					0.781
France	18	78.3	5	21.7		22	95.7	1	04.3		8	66.7	4	33.3		5	45.5	6	54.5	
Sweden	54	78.3	15	21.7		24	66.7	12	33.3		13	44.8	16	55.2		25	64.1	14	35.9	
Italy	14	70.0	6	30.0		10	66.7	5	33.3		2	22.2	7	77.8		8	57.1	6	42.9	
Germany	65	92.9	5	07.1		19	65.5	10	34.5		18	69.2	8	30.8		12	54.5	10	45.5	
Portugal	49	69.0	22	31.0		25	55.6	20	44.4		9	40.9	13	59.1		24	63.2	14	36.8	
Size of unit of residence (inhabitants)					0.444					0.629					0.601					0.479
>200,000	64	76.2	20	23.8		28	65.1	15	34.9		15	55.6	12	44.4		20	52.6	18	47.4	
3,000–200,000	94	78.3	26	21.7		50	65.8	26	34.2		22	45.8	26	54.2		40	64.5	22	35.5	
<3,000	41	85.4	7	14.6		21	75.0	7	25.0		13	56.5	10	43.5		13	56.5	10	43.5	
Lifestyle					0.776					0.862					0.535					0.219
Living alone	23	74.2	8	25.8		8	66.7	4	33.3		1	25.0	3	75.0		10	50.0	10	50.0	
Living accompanied	155	79.5	40	20.5		73	66.4	37	33.6		42	52.5	38	47.5		55	59.1	38	40.9	
In care facilities	21	80.8	5	19.2		18	72.0	7	28.0		6	46.2	7	53.8		9	81.8	2	18.2	
Education level					0.128					0.917					0.149					0.916
Did not complete secondary education	123	82.0	27	18.0		62	66.0	32	34.0		34	47.2	38	52.8		43	58.9	30	41.1	
Secondary education	49	79.0	13	21.0		28	70.0	12	30.0		11	55.0	9	45.0		18	56.2	14	43.8	
Tertiary education	24	66.7	12	33.3		7	70.0	3	30.0		4	100	0	00.0		10	62.5	6	37.5	
Parental education level					0.219					0.520					0.084					0.595
Did not complete secondary education	70	77.8	20	22.2		32	61.5	20	38.5		15	41.7	21	58.3		25	58.1	18	41.9	
Secondary education	71	84.5	13	15.5		31	67.4	15	32.6		21	67.7	10	32.3		21	53.8	18	46.2	
Tertiary education	55	73.3	20	26.7		34	72.3	13	27.7		14	46.7	16	53.3		26	65.0	14	35.0	
Wealth					0.599					0.996					0.483					0.278
No or minimal financial difficulties	148	80.0	37	20.0		66	67.3	32	32.7		33	49.3	34	50.7		56	61.5	35	38.5	
Financial difficulties	50	76.9	15	23.1		31	67.4	15	32.6		16	57.1	12	42.9		14	50.0	14	50.0	

SD, standard deviation; GMFCS, Gross Motor Function Classification System; FCCS, Fonctionnal Communication Classification System.

Bivariate analyses showed an interaction between the severity of gross motor dysfunction and the attitudinal environment in participants who reported a need for physiotherapy. The corresponding models were therefore run separately for individuals with GMFCS IV–V and those with GMFCS I–III. All models were adjusted as follows. In the beginning, all models were adjusted for sex and country of residence. Of the impairments and/or comorbidities, only walking ability was included in Models 1 with the exception of participants requiring speech therapy, for whom communication performance was also retained. Models 2 incorporated these factors, along with retained sociodemographic characteristics, specifically the population size of their unit of residence and lifestyle, without the addition of any other characteristics. The physical environment was never significantly associated with unmet healthcare needs, regardless of the intervention. Conversely, the social environment was associated to varying degrees of unmet healthcare needs in all four interventions. When one unmet need for the social environmental was reported, the odds of also reporting an unmet need for physiotherapy increased more than twofold (OR 2.5; 95% CI: 1.18–5.55). When 2–4 unmet social environmental needs were reported, the odds of reporting unmet occupational therapy and psychological counselling needs were OR 6.58 (95% CI: 1.17–41.38) and 12.89 (95% CI: 2.14–120.91), respectively. A significant trend was observed for speech therapy. With regards to the attitudinal environment, when at least one unmet environmental need was reported, the odds of also reporting an unmet healthcare need increased more than three-fold for speech therapy (OR 3.68; 95% CI: 1.41–10.31) and for physiotherapy (OR 3.77; 95% CI: 1.22–12.80), the latter only in those with severe motor impairment (GMFCS IV–V) (Table 4).

**Table 4:** Association between the adequacy of EF and unmet health needs in the 4 domains.

	Unmet physiotherapy need (*n* = 253)					Unmet occupational therapy need (*n* = 148)					Unmet speech therapy need (*n* = 98)					Unmet psychological counselling need (*n* = 124)				
		Model 1^a^		Model 2^b^			Model 1^a^		Model 2^b^			Model 1^a^		Model 2^b^			Model 1^c^		Model 2^d^	
EF (unmet needs)	*n*	OR	95% CI	OR	95% CI	*n*	OR	95% CI	OR	95% CI	*n*	OR	95% CI	OR	95% CI	*n*	OR	95% CI	OR	95% CI
Physical	** **	** **	** **	** **	** **	** **	** **	** **	** **	** **	** **	** **	** **	** **	** **	** **	** **	** **	** **	** **
0	75	1.00	** **	1.00	** **	41	1.00	** **	1.00	** **	25	1.00	** **	1.00	** **	41	1.00	** **	1.00	** **
1–3	101	0.48	(0.21–1.07)	0.50	(0.22–1.11)	59	0.49	(0.19–1.25)	0.53	(0.19–1.41)	39	0.80	(0.25–2.54)	0.82	(0.25–2.68)	51	0.71	(0.26–1.96)	0.89	(0.31–2.54)
4–9	72	0.92	(0.37–2.31)	0.89	(0.35–2.25)	45	0.57	(0.19–1.72)	0.54	(0.17–1.66)	31	2.05	(0.52–8.73)	1.95	(0.47–8.63)	31	1.50	(0.42–5.57)	1.77	(0.46–7.16)
Social		** **	** **	** **	** **	** **	** **	** **	** **	** **	** **	** **	** **	** **	** **	** **	** **	** **	** **	** **
0	172	1.00	** **	1.00	** **	98	1.00	** **	1.00	** **	62	01.00	** **	1.00	** **	83	01.00	** **	01.00	** **
1	62	2.64	(1.24–5.69)^†^	2.55	(1.18–5.55)^†^	38	2.50	(0.99–6.68)	2.52	(0.98–6.83)	29	04.23	(1.40–14.48)^†^	4.07	(1.32–14.09)^†^	30	01.88	(0.68–5.38)	02.08	(0.71–6.39)
2–4	15	2.59	(0.64–9.09)	2.95	(0.71–10.59)	8	6.09	(1.10–36.86)^†^	6.58	(1.17–41.38)^†^	6	12.10	(1.58–255.71)^†^	9.91	(1.22–214.86)^†^	10	13.58	(2.39–120.41)^†^	12.89	(2.14–120.91)^†^
Attitudinal (all individuals)						** **	** **	** **	** **	** **	** **	** **	** **	** **	** **	** **	** **	** **	** **	** **
0		** **	** **	** **	** **	88	1.00	** **	1.00	** **	58	1.00	** **	1.00	** **	73	1.00	** **	1.00	** **
1–3		** **	** **	** **	** **	56	1.41	(0.64–3.10)	1.28	(0.57–2.88)	37	3.25	(1.30–8.62)^†^	3.68	(1.41–10.31)^†^	47	0.82	(0.35–1.89)	0,94	(0.39–2.28)
Attitudinal (GMFCS I–II–III)						** **	** **	** **	** **	** **	** **	** **	** **	** **	** **	** **	** **	** **	** **	** **
0	99	1.00	** **	1.00	** **	** **	** **	** **	** **	** **	** **	** **	** **	** **	** **	** **	** **	** **	** **	** **
1–3	40	0.58	(0.21–1.51)	0.54	(0.19–1.45)	** **	** **	** **	** **	** **	** **	** **	** **	** **	** **	** **	** **	** **	** **	** **
Attitudinal (GMFCS IV–V)						** **	** **	** **	** **	** **	** **	** **	** **	** **	** **	** **	** **	** **	** **	** **
0	64	1.00		1.00		** **	** **	** **	** **	** **	** **	** **	** **	** **	** **	** **	** **	** **	** **	** **
1–3	43	3.84	(1.27–12.88)^†^	3.77	(1.22–12.80)^†^	** **	** **	** **	** **	** **	** **	** **	** **	** **	** **	** **	** **	** **	** **	** **

EF, environmental factors; GMFCS, Gross Motor Functional Classification System.

^a^ adjusted for country, sex and GMFCS.

^b^ adjusted for country, sex, GMFCS and sociodemographic characteristics (size of unit of residence and lifestyle).

^c^ adjusted for country, sex, GMFCS and FCCS.

^d^ adjusted for country, sex, GMFCS, FCCS and sociodemographic characteristics (size of unit of residence and lifestyle).

^†^ 95% CI excluding one.

The results did not change after excluding individuals with proxy-reports or excluding participants from each country one by one.

## Discussion

### Key findings

Our study showed that the commonest healthcare need was physiotherapy, which was reported by more than four out of five young adults with CP. Among those who expressed a need for care, the proportion whose need was not met varied according to the type of care required: 20.8%–48.5% for rehabilitation, and 40.3% for psychological counselling. We found an association between an environment inadequate to the specific needs of young people with CP and their care needs. More specifically, the accumulation of unmet needs in the social environment, exploring support from the personal assistant, family and friends, healthcare staff and colleagues, and strangers, was associated with unmet needs in all health domains explored, even taking account of the severity of impairments and the socio-demographic characteristics of the individuals. The lack of supportive attitudes increased by more than 3-fold the odds of also reporting an unmet need for speech therapy and physiotherapy, only among those with the most severely impaired gross motor function in the latter case. Finally, our study showed no association between unmet needs in the physical environment and unmet healthcare needs.

### Strengths and limitations

This cross-sectional study enrolled 310 young adults with CP, making it one of the largest studies ever conducted in this population. We identified cases either from population-based registries or from several independent sources, using the same definition of CP, which limited case selection and classification errors. Nevertheless, recruitment in Germany and Portugal, although based on a variety of sources, included rehabilitation centres, hospitals and specialized institutes, which may first result in a lack of sample representativeness, and reduce the proportion of individuals with unmet healthcare needs. It was important to take this potential selection bias into account, given that participants from these two countries represented more than half of our sample (56.8%).

We performed the analyses by pooling self-reported and proxy-reported data to maximize the inclusion in the study of severely impaired people who are not usually included in this type of studies. Although we considered that, irrespective of the respondent, self-report was the best available estimate of environmental adequacy and unmet healthcare needs, we cannot rule out underestimation or overestimation of both these pieces of information when using proxy-reports (24, 25). Nevertheless, our findings did not change when we excluded proxy-reports in our sensitivity analyses.

The measurement of the adequacy of the environment to the specific needs of the target population was based on the EAEQ, a questionnaire developed as part of the SPARCLE study to provide a comprehensive assessment in line with the ICF. As part of this exploratory study, we made choices that respected the contours of this approach. First, we selected EAEQ items in the physical (accessibility of facilities and transport), social and attitudinal environment that were important for access to care. Based on the assumption that the accumulation of unmet needs in the environment was associated with unmet healthcare needs, we summed the items in each environmental domain to create one variable by domain quantifying environmental adequacy. However, due to the low prevalence of unmet needs for some elements of the environment and the lack of existing studies to guide us in our grouping choices, we opted for a graphical categorization. This choice enabled to obtain a sufficient number of individuals per category (at least five), except for the attitudinal domain, which limited the analysis of a potential cumulative effect. Another limitation was that this method assigned the same weight to each EF, which may potentially minimize or maximize their relative contribution (26).

### Interpretation

To date, relatively little research has explored the healthcare needs of young adults with CP, focusing more on the utilization of healthcare services. A literature review by Manikandan et al. (21) of 57 studies involving 14,300 adults (mean age 18–48 years) found that 44% had consulted a physiotherapist, 27% an occupational therapist, 16% a speech therapist, and 11% a psychologist or psychiatrist in the past year. Although we did not have a direct measure of health service use, we observed similar frequencies when looking at the proportion of met healthcare needs for occupational therapy and speech therapy (32%; *n* = 100/310% and 16%; *n* = 50/310, respectively), while it was higher for physiotherapy (65%; *n* = 200/310) and psychological counselling (24%; *n* = 74/310). The high proportion of participants from Germany, where the number of physiotherapists is relatively high (244 physical therapists per 100,000 inhabitants (27) compared with 144 per 100,000 in France (28), for instance) may partly explain this difference.

While the utilization of health services is an important piece of information, measuring the need for these services and assessing how often they are not met are crucial in studying the factors limiting access. We found that, in descending order of frequency, the health care needs expressed were 81.6% for physiotherapy, 47.7% for occupational therapy, 40.0% for psychological counselling and 31.6% for speech therapy. To our knowledge, no recent study has comparable data. A cross-sectional study in Ireland of 40 young people aged 16–22 in transition to adult services found that 67.5% expressed healthcare needs related to mobility (which may partly correlate with physiotherapy), 50% related to positioning (which may partly correlate with occupational therapy), and 26.3% related to speech (likely correlated with speech therapy) (29). Another Anglo-Irish study of 106 14–18 year olds found similar results, with 58.4% of needs related to mobility, 25.5% to positioning, and 21.7% to speech (24).

In the literature, speech therapy has been described as the profession with the highest proportion of unmet needs, ranging from 39% to 70% (24, 29). Conversely, physiotherapy had the lowest proportion (12%−44%) (24, 29). Our results are consistent with these observations. In our study, severe motor impairment (GMFCS IV–V) was associated with better satisfaction of healthcare needs in the four professions, which differs from the data in the literature (9, 24) which showed that the severity of the disability tended to limit access to rehabilitation and psychological counselling. With regard to socio-demographic factors, an American study showed that adult men with disabilities were less likely than women to report at least one unmet healthcare need (60.7% vs. 75.7%) (30). However, we did not find this in our sample, and even found the opposite result for psychological counselling. A French study showed that living in care facilities was associated with a reduction in unmet healthcare needs (23), which was not observed in our study.

To our best knowledge, no publication has specifically evaluated the relationship between unmet healthcare needs of young adults with CP and the adequacy of their environment to meet their specific needs. Therefore, our interpretations are based solely on the results of our study, and we only have the opportunity to put forward a few hypotheses to explain this relationship. Our results highlight the importance of the social environment and support. At this age, greater independence and emancipation, particularly from parents, could partially explain why young adults have greater unmet healthcare needs than children. It may be hypothesized that the lack of association between the physical environment and unmet healthcare needs is due to the fact that the physical environment is often compensated by social support and assistance from family and friends. Thus, a young adult could move around easily if accompanied, even in a less suitable environment, whereas an individual with less support would have more difficulty even in a better adapted physical environment. It is also conceivable that this difference might be liked to cognitive impairments, which often pose more challenges for independent mobility than motor impairments, for which adaptations are usually possible. Our findings suggest that the greater the unmet needs in the social environment, the greater the unmet healthcare needs for occupational therapy, speech therapy, and psychological counselling. However, due to the small number of participants with multiple unmet needs in their social environment, questions arise regarding these concepts, their content, and their implications for access to care for individuals with CP. Consequently, these findings should be interpreted with caution. Our findings indicated that attitudinal support was associated with unmet healthcare needs for speech therapy, which affects most people with severe motor and cognitive impairments, and for physiotherapy in people with severe motor impairments. This suggests that this support is particularly crucial to ensure access to healthcare for these therapies in severely impaired young adults with CP. We did not find this association in individuals who required occupational therapy or psychological counselling.

## Conclusion

Our study showed that the adequacy of the environment, both social and attitudinal, can have an impact on unmet healthcare needs in different therapeutic areas in young adults with CP. It sheds valuable light on the factors influencing unmet health needs in this population. However, further research is needed to better understand and delineate these two environmental domains and deeper explore their relationship with access to healthcare.

## Data Availability

The raw data supporting the conclusions of this article will be made available by the authors, without undue reservation.

## SPARCLE 3 collaborative group

Joaquim J. M. Alvarelhão, Jérôme Fauconnier, Kate Himmelmann, Marco Marcelli, Silke Schmidt and Ute Thyen.
