# Supplementary material for: Unmet environmental needs and unmet healthcare needs in a population of young adults with cerebral palsy: what the SPARCLE study tells us
Source: Front Rehabil Sci. 2024 Feb 2;5:1294999. doi: 10.3389/fresc.2024.1294999 (PMC10869570; doi:10.3389/fresc.2024.1294999)
Supplement: Supplementary file 1 [file Table1.docx]

**TABLE S1** Items from the EAEQ *a priori* related to the environmental needs for healthcare access

|  |  |  |  |
| --- | --- | --- | --- |
|  | **Physical environment** |  |  |
|  | Ramps in public places | Need or not? If needed, available or not? |  |
|  | Lifts / escalators | Need or not? If needed, available or not? |  |
|  | Adapted doorways | Need or not? If needed, available or not? |  |
|  | Accessible pavements in your town | Need or not? If needed, available or not? |  |
|  | Modified wheelchair | Need or not? If needed, available or not? |  |
|  | Adapted vehicle for getting around | Need or not? If needed, available or not? |  |
|  | Accessible car parking | Need or not? If needed, available or not? |  |
|  | Adequate public transport | Need or not? If needed, available or not? |  |
|  | Accessible public transport | Need or not? If needed, available or not? |  |
|  |  |  |  |
|  | **Social environment** |  |  |
|  | Personal assistant | Need or not? If needed, available or not? |  |
|  | Assistance from family/friends | Need or not? If needed, available or not? |  |
|  | Assistance from healthcare staff/colleagues | Need or not? If needed, available or not? |  |
|  | Assistance from strangers | Need or not? If needed, available or not? |  |
|  |  |  |  |
|  | **Attitudinal environment** |  |  |
|  | Positive attitude from family/friends | Available or not? |  |
|  | Positive attitude from healthcare staff/colleagues | Available or not? |  |
|  | Positive attitude from strangers | Available or not? |  |
|  |  |  |  |
