# Supplementary material for: Unmet environmental needs and unmet healthcare needs in a population of young adults with cerebral palsy: what the SPARCLE study tells us
Source: Front Rehabil Sci. 2024 Feb 2;5:1294999. doi: 10.3389/fresc.2024.1294999 (PMC10869570; doi:10.3389/fresc.2024.1294999)
Supplement: Supplementary file 2 [file Table2.docx]

**TABLE S2** Description of responses for each environmental item among young adults in the overall sample and by health needs subgroups

|  |  | **All participants**  **(n = 310)** | | **Physiotherapy**  **need**  **(n = 253)** | | **Occupational therapy need**  **(n = 148)** | | **Speech therapy need**  **(n = 98)** | | **Psychological counselling need**  **(n = 124)** | |  |
| --- | --- | --- | --- | --- | --- | --- | --- | --- | --- | --- | --- | --- |
|  |  |  |  |  |  |  |  |  |  |  |  |  |
|  | **Physical environment (9 items)** | **N** | **%** | **n** | **%** | **n** | **%** | **n** | **%** | **n** | **%** |  |
|  |  |  |  |  |  |  |  |  |  |  |  |  |
|  | **Ramps in public places** |  |  |  |  |  |  |  |  |  |  |  |
|  | Not needed | 145 | 47.2 | 96 | 38.2 | 46 | 31.3 | 31 | 32.3 | 58 | 46.8 |  |
|  | Needed and available | 55 | 17.9 | 54 | 21.5 | 34 | 23.1 | 27 | 28.1 | 20 | 16.1 |  |
|  | Needed and not available | 107 | 34.9 | 101 | 40.2 | 67 | 45.6 | 38 | 39.6 | 46 | 37.1 |  |
|  | **Lifts / escalators** |  |  |  |  |  |  |  |  |  |  |  |
|  | Not needed | 129 | 41.6 | 82 | 32.4 | 40 | 27.0 | 29 | 29.6 | 52 | 41.9 |  |
|  | Needed and available | 113 | 36.5 | 107 | 42.3 | 71 | 48.0 | 43 | 43.9 | 48 | 38.7 |  |
|  | Needed and not available | 68 | 21.9 | 64 | 25.3 | 37 | 25.0 | 26 | 26.5 | 24 | 19.4 |  |
|  | **Adapted doorways** |  |  |  |  |  |  |  |  |  |  |  |
|  | Not needed | 169 | 54.5 | 121 | 47.8 | 55 | 37.2 | 37 | 37.8 | 67 | 54.0 |  |
|  | Needed and available | 69 | 22.3 | 64 | 25.3 | 48 | 32.4 | 30 | 30.6 | 24 | 19.4 |  |
|  | Needed and not available | 72 | 23.2 | 68 | 26.9 | 45 | 30.4 | 31 | 31.6 | 33 | 26.6 |  |
|  | **Accessible pavements in your town** |  |  |  |  |  |  |  |  |  |  |  |
|  | Not needed | 139 | 45.0 | 97 | 38.5 | 49 | 33.3 | 31 | 32.0 | 59 | 47.6 |  |
|  | Needed and available | 69 | 22.3 | 64 | 25.4 | 37 | 25.2 | 25 | 25.8 | 23 | 18.5 |  |
|  | Needed and not available | 101 | 32.7 | 91 | 36.1 | 61 | 41.5 | 41 | 42.3 | 42 | 33.9 |  |
|  | **Modified wheelchair** |  |  |  |  |  |  |  |  |  |  |  |
|  | Not needed | 154 | 49.7 | 105 | 41.5 | 50 | 33.8 | 34 | 34.7 | 66 | 53.2 |  |
|  | Needed and available | 142 | 45.8 | 136 | 53.8 | 92 | 62.2 | 61 | 62.2 | 52 | 41.9 |  |
|  | Needed and not available | 14 | 04.5 | 12 | 04.7 | 6 | 04.1 | 3 | 03.1 | 6 | 04.8 |  |
|  | **Adapted vehicle for getting around** |  |  |  |  |  |  |  |  |  |  |  |
|  | Not needed | 138 | 44.5 | 95 | 37.5 | 58 | 39.2 | 38 | 38.8 | 62 | 50.0 |  |
|  | Needed and available | 110 | 35.5 | 100 | 39.5 | 60 | 40.5 | 40 | 40.8 | 31 | 25.0 |  |
|  | Needed and not available | 62 | 20.0 | 58 | 22.9 | 30 | 20.3 | 20 | 20.4 | 31 | 25.0 |  |
|  | **Accessible car parking** |  |  |  |  |  |  |  |  |  |  |  |
|  | Not needed | 112 | 36.2 | 74 | 29.4 | 38 | 25.7 | 23 | 23.7 | 46 | 37.1 |  |
|  | Needed and available | 114 | 36.9 | 103 | 40.9 | 63 | 42.6 | 38 | 39.2 | 46 | 37.1 |  |
|  | Needed and not available | 83 | 26.9 | 75 | 29.8 | 47 | 31.8 | 36 | 37.1 | 32 | 25.8 |  |
|  | **Adequate public transport** |  |  |  |  |  |  |  |  |  |  |  |
|  | Not needed | 139 | 45.0 | 104 | 41.3 | 65 | 43.9 | 46 | 46.9 | 47 | 37.9 |  |
|  | Needed and available | 112 | 36.2 | 93 | 36.9 | 49 | 33.3 | 29 | 29.6 | 45 | 36.3 |  |
|  | Needed and not available | 58 | 18.8 | 55 | 21.8 | 34 | 23.0 | 23 | 23.5 | 32 | 25.8 |  |
|  | **Accessible public transport** |  |  |  |  |  |  |  |  |  |  |  |
|  | Not needed | 147 | 47.7 | 105 | 41.8 | 63 | 42.9 | 41 | 42.3 | 53 | 42.7 |  |
|  | Needed and available | 99 | 32.1 | 88 | 35.1 | 47 | 32.0 | 29 | 29.9 | 42 | 33.9 |  |
|  | Needed and not available | 62 | 20.1 | 58 | 23.1 | 37 | 25.2 | 27 | 27.8 | 29 | 23.4 |  |
|  |  |  |  |  |  |  |  |  |  |  |  |  |
|  | **Social environment (4 items)** | **N** | **%** | **n** | **%** | **n** | **%** | **n** | **%** | **n** | **%** |  |
|  |  |  |  | 44 (17.4%) |  |  |  |  |  |  |  |  |
|  | **Personal assistant** |  |  |  |  |  |  |  |  |  |  |  |
|  | Not needed | 185 | 59.7 | 134 | 53.0 | 67 | 45.3 | 36 | 36.7 | 70 | 56.5 |  |
|  | Needed and available | 77 | 24.8 | 75 | 29.6 | 57 | 38.5 | 40 | 40.8 | 34 | 27.4 |  |
|  | Needed and not available | 48 | 15.5 | 44 | 17.4 | 24 | 16.2 | 22 | 22.4 | 20 | 16.1 |  |
|  | **Assistance from family/friends** |  |  |  |  |  |  |  |  |  |  |  |
|  | Not needed | 63 | 20.4 | 37 | 14.7 | 14 | 09.5 | 5 | 05.1 | 21 | 16.9 |  |
|  | Needed and available | 238 | 77.0 | 207 | 82.1 | 127 | 86.4 | 90 | 91.8 | 97 | 78.2 |  |
|  | Needed and not available | 8 | 02.6 | 8 | 03.2 | 6 | 04.1 | 3 | 03.1 | 6 | 04.8 |  |
|  | **Assistance from healthcare staff/colleagues** |  |  |  |  |  |  |  |  |  |  |  |
|  | Not needed | 89 | 28.8 | 57 | 22.6 | 24 | 16.3 | 15 | 15.3 | 29 | 23.4 |  |
|  | Needed and available | 209 | 67.6 | 184 | 73.0 | 118 | 80.3 | 80 | 81.6 | 87 | 70.2 |  |
|  | Needed and not available | 11 | 03.6 | 11 | 04.4 | 5 | 03.4 | 3 | 03.1 | 8 | 06.5 |  |
|  | **Assistance from strangers** |  |  |  |  |  |  |  |  |  |  |  |
|  | Not needed | 158 | 51.0 | 112 | 44.3 | 59 | 39.9 | 38 | 38.8 | 56 | 45.2 |  |
|  | Needed and available | 115 | 37.1 | 106 | 41.9 | 65 | 43.9 | 44 | 44.9 | 49 | 39.5 |  |
|  | Needed and not available | 37 | 11.9 | 35 | 13.8 | 24 | 16.2 | 16 | 16.3 | 19 | 15.3 |  |
|  |  |  |  |  |  |  |  |  |  |  |  |  |
|  | **Attitudinal environment (3 items)** | **N** | **%** | **n** | **%** | **n** | **%** | **n** | **%** | **n** | **%** |  |
|  |  |  |  |  |  |  |  |  |  |  |  |  |
|  | **Positive attitude from family/friends** |  |  |  |  |  |  |  |  |  |  |  |
|  | Yes | 302 | 97.4 | 245 | 96.8 | 142 | 95.9 | 96 | 98.0 | 118 | 95.2 |  |
|  | No | 8 | 02.6 | 8 | 03.2 | 6 | 04.1 | 2 | 02.0 | 6 | 04.8 |  |
|  | **Positive attitude from healthcare staff/colleagues** |  |  |  |  |  |  |  |  |  |  |  |
|  | Yes | 293 | 95.1 | 236 | 94.0 | 138 | 93.2 | 91 | 93.8 | 115 | 93.5 |  |
|  | No | 15 | 04.9 | 15 | 06.0 | 10 | 06.8 | 6 | 06.2 | 8 | 06.5 |  |
|  | **Positive attitude from strangers** |  |  |  |  |  |  |  |  |  |  |  |
|  | Yes | 218 | 71.5 | 179 | 71.6 | 100 | 68.5 | 64 | 66.0 | 82 | 67.2 |  |
|  | No | 87 | 28.5 | 71 | 28.4 | 46 | 31.5 | 33 | 34.0 | 40 | 32.8 |  |
|  |  |  |  |  |  |  |  |  |  |  |  |  |
